# Transcriptomic and functional profiling of *Acinetobacter baumannii* reveals adaptation to burn patient blood and time-dependent responses to human serum

**DOI:** 10.3389/fmicb.2025.1635690

**Published:** 2025-08-29

**Authors:** Hannaneh Ghavanloughajar, Amanda M. V. Brown, Amrika Deonarine, Yongli Wang, Abdul N. Hamood

**Affiliations:** ^1^Department of Biological Sciences, Texas Tech University, Lubbock, TX, United States; ^2^Department of Civil, Environmental, and Construction Engineering, Texas Tech University, Lubbock, TX, United States; ^3^Department of Immunology and Molecular Microbiology, Texas Tech University Health Sciences Center, Lubbock, TX, United States; ^4^Department of Surgery, Texas Tech University Health Sciences Center, Lubbock, TX, United States

**Keywords:** host-pathogen interaction, *Acinetobacter baumannii*, human whole blood, burn patients, immunocompromised patients, RNA-Seq analysis, human serum, acinetobactin

## Abstract

*Acinetobacter baumannii* is a critical threat to immunocompromised patients, particularly those with burn injuries. Despite its clinical significance, little is known about how this bacterium adapts to the complex environment of burn patient blood, which differs significantly from that of healthy individuals. To address this, our methods included analyses of a blood-isolated strain of *A. baumannii* A118 grown ex vivo in whole blood from healthy volunteers (WBHV) and burn patients (WBBP). Transcriptomic analysis revealed host-specific expression patterns, including the downregulation of acinetobactin siderophore genes in WBBP—suggesting increased free iron levels—and the strong upregulation of nitrate/nitrite metabolism genes, indicating altered nitrogen availability in burn patients’ blood. Since serum makes up the majority of blood volume and contains key factors influencing bacterial physiology, we examined the transcriptomic response of *A. baumannii* to pooled human serum in an LB + 10% serum model at two different time points of growth, isolating the impact of soluble components free from immune cells and building on patterns seen in whole blood. Consistent with our ex vivo findings, we again observed dynamic regulation of the acinetobactin operon—this time in response to serum exposure: an initial upregulation of key iron uptake genes at early time points was followed by strong downregulation later, suggesting a transient iron starvation response that is modulated as intracellular iron accumulates, which can be supported by our ICP-MS results, revealing increased intracellular iron and other metal ions in serum-treated bacteria over time. To support additional serum-induced transcriptomic findings beyond acinetobactin, we used multiple experimental approaches: LC–MS/MS of outer membrane protein (OMP) extracts identified a serum-upregulated acinetobactin transporter; and phenotypic assays revealed that serum enhanced biofilm formation, increased twitching motility, elevated mortality in the *Galleria mellonella* infection model, and raised minimum inhibitory concentration (MIC) for multiple antibiotics. In conclusion, these findings expand our understanding of pathogen behavior in clinically relevant conditions and suggest that host-specific blood physiology—especially in burn patients—can shape the course of infection.

## 1 Introduction

*Acinetobacter baumannii* is a Gram-negative, non-fermenting opportunistic pathogen commonly associated with hospital-acquired infections, particularly in immunocompromised patients in intensive care units. It is also a major cause of morbidity and mortality among hospitalized burn patients (Albrecht et al., 2006; Vahdani et al., 2012). *A. baumannii* exhibits high genomic plasticity, enabling rapid acquisition of antibiotic resistance and adaptation to a wide range of environmental stimuli (Murray et al., 2017). While individual blood components have been studied for their impact on the gene expression of *A. baumannii* (Quinn et al., 2018; Martinez et al., 2021; Pimentel et al., 2021), the direct transcriptomic response to human whole blood—particularly in comparison to the complex and distinct environment of burn patient blood—remains largely unexplored. Multiple studies have indicated that hospitalized burn patients are especially vulnerable to *A. baumannii* infections, with the bacterium reported as the second most common cause of hospital-acquired infections in this population (Owlia et al., 2012; Almasaudi, 2018). Additionally, outbreaks of *A. baumannii* frequently occur in intensive care units (ICUs) and burn wards (Munier et al., 2017). Despite the clinical relevance, little is known about how this highly adaptable nosocomial pathogen, responds at the gene expression level to such physiologically diverse blood environments.

To address this gap in knowledge, we employed RNA-Seq to compare the global gene expression profiles of *A. baumannii* A118—a blood isolate from an ICU patient—grown ex vivo in whole blood from burn patients (WBBP) versus that from healthy volunteers (WBHV).

Next, to explore the specific impact of serum-derived factors on bacterial gene regulation, we extended our investigation to pooled human serum—an abundant and clinically relevant component of blood. Several *A. baumannii* strains, including A118, have been shown to grow in the presence of non–heat-inactivated human serum (Pimentel et al., 2021). Using RNA-Seq, we analyzed the transcriptomic response of *A. baumannii* A118 grown in LB + 10% serum compared to LB alone, capturing changes at both logarithmic and late growth phases. This time-resolved approach allowed us to assess not only serum-induced gene expression, but also how these responses shift with bacterial growth. Although previous studies have examined serum effects on a limited set of genes using qRT-PCR (Pimentel et al., 2021), a comprehensive transcriptomic analysis of *A. baumannii* under serum exposure has not, to our knowledge, been performed. Our study fills this gap by uncovering broad, time-dependent regulatory patterns and offering new insights into how this major component of blood shapes bacterial physiology throughout the course of infection.

## 2 Materials and methods

### 2.1 Ethics statement for collection of whole blood

This study (RB number L21-156 and L13-140) were approved by the Texas Tech University Health Sciences Center Lubbock Institutional Review Board on 12/07/2021 and 07/23/2014, respectively and remained open until all study activities were completed. Informed written consent was obtained from healthy volunteers (HVs) and burn patients (BPs) at University Medical Center, Lubbock, TX, in compliance with ethical practices. HVs had no acute or chronic medical conditions and all subjects were aged 18–89. Blood samples were collected by CRI staff through venipuncture as per the IRB-approved protocol. Blood was collected within 72 h of admission. A total of 25 mL of blood was collected from each person into three BD Vacutainer tubes (Becton Dickinson, NJ, USA), containing 0.35% sodium polyanethole sulfonate as an anticoagulant in 1.7 mL of 0.85% sodium chloride (SPS). Per the IRB protocol, blood samples were de-identified and were given unique numbers by the CRI staff before the samples were sent to the research laboratory. All methods performed on the samples were in compliance with the relevant guidelines and regulations of the IRB-approved protocol.

### 2.2 Bacterial strain, culture conditions and growth curves

*Acinetobacter baumannii* strain A118 was received from ATCC and used in all experiments. This strain was isolated from a blood culture of an intensive care unit patient in 1995 in Buenos Aires City, Argentina [18]. Bacteria were routinely cultured overnight in Luria-Bertani (LB) broth at 37°C with shaking prior to subculturing them into whole blood samples, LB, or LB supplemented with 10% of pooled human serum (LB + 10% serum) (MP Biomedical, CA, USA) in 1:100 ratio. In growth curve assays, bacterial growth of 3 independent replicates was monitored by measuring colony-forming units (CFU) every 2 h over an 8-h period. For bacterial RNA extraction from LB and LB + 10% serum cultures, samples were taken from three individual replicates per condition at 4 and 8 h post inoculation (HPI). The samples were then pelleted, resuspended in LB, treated with RNAprotect (Qiagen, Germany), and subsequently processed for bacterial RNA extraction.

### 2.3 Growth of *Acinetobacter baumannii* A118 in whole blood and subsequent bacterial isolation

Whole blood from three BD Vacutainer tubes of each subject (HV or BP) was pooled to minimize RBC lysis. Aliquots of 7.5 mL were distributed into three flasks as technical replicates, inoculated with overnight *A. baumannii* A118 LB culture as previously mentioned, and incubated at 37°C. After 4 h, when bacterial growth reached ~10^8^ CFU/mL, samples were diluted 1:1 with 1X PBS and layered over lymphocyte separation medium (Lonza, Switzerland) and were centrifuged according to the manufacturer’s protocol. Then, the layers containing lymphocytes and granulocytes were discarded, while the RBC and bacterial pellet layer was saved and treated repeatedly with erythrocyte lysis buffer (Qiagen, Germany) and was centrifuged according to the manufacturer’s protocol until all RBCs were lysed and only bacterial cells remained. The pellet was resuspended in LB, treated with RNAprotect (Qiagen, Germany), and then processed for bacterial RNA extraction.

### 2.4 Bacterial RNA extraction and sequencing

Bacterial RNA was extracted using the RNeasy Mini Kit (Qiagen, Germany) according to the manufacturer’s recommendations followed by an additional on-column DNase treatment to eliminate any remaining traces of genomic DNA. Total RNA concentration and integrity were determined by Nanodrop spectrophotometer (Nanodrop Technologies, DE, USA) and TapeStation 2,200 (Agilent, CA, USA), respectively. rRNA depletion was achieved using NEB Next rRNA Depletion Kit (Human/Mouse/Rat) (New England Biolabs, MA, USA). RNA fragmentation, double stranded cDNA synthesis and adaptor ligation were carried out using NEBNext Ultra II Directional RNA Library Prep according to the manufacture protocol (New England Biolabs, MA, USA). PCR enriched libraries were quantified using picogreen (Thermofisher scientific, MA, USA) and equimolar indexed libraries were pooled. Pooled libraries were assessed using the Agilent Tapestation 2,400 (Agilent, CA, USA). The libraries were then diluted to 250 pM, spiked with 1% phiX libraries (Illumina control), and sequenced using Illumina NovaSeq 6,000 (Illumina, CA, USA).

### 2.5 RNA-Seq data analysis

Paired-end reads of each sample were mapped to the *A. baumannii* A118 reference transcriptome (NCBI RefSeq assembly: ASM1467273v1) by the pseudo-alignment-based tool, Kallisto (Bray et al., 2016) with 100 bootstraps per sample for quantification of transcript sequences. Then, Kallisto output files were imported to R software (version 4.2.1) (R Core Team, R, 2013) and DESeq2 (version 1.36.0) (Love et al., 2014) was used to normalize transcript counts and test for differential gene expression by the Wald test after integrating the dispersion estimate. The *p*-values were corrected for multiple testing using the Benjamini–Hochberg procedure (Benjamini and Hochberg, 1995). Differentially expressed genes (DEGs) were identified based on their corresponding adjusted p-value (padj ≤ 0.05 and |log2foldchange| ≥ 1).

### 2.6 Gene ontology enrichment analysis

GO analysis of DEGs was performed by STRING^1^ (Szklarczyk et al., 2019). False Discovery Rate (FDR) describes the significance of the enrichment, with *p*-values adjusted for multiple testing within each category using the Benjamini–Hochberg procedure. Strength values were calculated by observed/expected ratio. Signal values were calculated as a weighted harmonic mean between the observed/expected ratio and -log(FDR). The FDR emphasizes larger terms, as they are more likely to yield lower p-values, whereas the strength value gives prominence to smaller terms, which often have a high foreground-to-background ratio but cannot achieve low FDR values due to their limited size. However, signal parameter aims to balance these two metrics, creating a more intuitive ranking of enriched terms (von Mering et al., 2005). Therefore, we selected it as the primary metric for displaying GO terms in our graphs. The other key enrichment display setting parameter defined as term similarity by STRING, filters terms using the Jaccard index to measure similarity between gene sets within a category (Szklarczyk et al., 2025). Terms were prioritized by *p*-value, and those exceeding a user-defined similarity threshold were excluded. The enrichment display settings for our analysis were adjusted as follows: Term similarity ≥0.70, FDR ≤ 0.05, Signal ≥ 0.50, Strength ≥ 0.25, and Minimum count in the network was set to 10. For network construction, we used the Full STRING network as the network type, selecting all available options for active interaction sources. The minimum required interaction score was set to 0.4, and the maximum number of interactions to show for both the first and second shell was set to none.

### 2.7 Quantitative analysis of biofilm formation

Biofilm formation of bacteria was assessed as previously described (Hammond et al., 2011; Alqahtani et al., 2021) with some modifications. Briefly, standardized bacterial cultures of *A. baumannii* A118 and *P. aeruginosa* PAO1 were prepared based on OD_600_ measurements. Then, 10 μL of each culture was added to each well of a 24-well plate containing 1 mL of fresh LB or LB containing different percentages of serum, ranging from 5 to 20% depending on the experiment. One sterile 6 mm cellulose disk (Becton Dickinson and Company, MD, USA) was placed in each well, and plates were incubated at 37°C shaking incubator for 24 h. After incubation, to determine the number of CFUs present in the biofilm, the disks were removed with sterile forceps, rinsed twice with 1x PBS to remove planktonic cells and were placed individually in microtubes containing 1 mL of 1x PBS. The microtubes were vortexed vigorously for 1 min to dislodge biofilms from the disks and CFU counts were then measured. Each experiment was performed in triplicates.

### 2.8 Antimicrobial susceptibility testing

Antimicrobial susceptibility testing was conducted as previously described (Huang et al., 2020; Le et al., 2021) with some modifications following the procedures recommended by the CLSI (Wikler, 2006). After OD_600_ adjustment, 100 μL of bacterial LB cultures were inoculated on Mueller-Hinton agar plates (MH) (Sigma-Aldrich, MA, USA) with and without 10% pooled human serum. Antimicrobial commercial E-strips (Liofilchem S.r.l., Italy) for Chloramphenicol (C), Imipenem (IMI), Amikacin (AK), Rifampicin (RD) and Tobramycin (TOB) were aseptically placed onto individual plates. Then, plates were incubated at 37°C for 18 h. Each antibiotic was tested in three replicates. Minimum inhibitory concentration (MIC) readings were performed according to the manufacturer’s protocol.

### 2.9 Isolation of bacterial OMPs, SDS-PAGE and LC–MS/MS protein identification

The OMPs extraction procedure was followed as previously described (Beasley et al., 2020) with some minor modifications. LB and LB + 10% serum were inoculated with *A. baumannii* A118 as previously mentioned and grown for 4 h. Then, cultures were centrifuged and cells were washed with sterile water and lysed by sonication (10 min pulses at 50% power 3 times) using a Kinematica Polytron P10-35 PCU-11 homogenizer (Kinematica AG, Switzerland). Lysed cells were centrifuged for 20 min at 18,450 × g to obtain a pellet containing both inner and outer membrane proteins. This pellet was resuspended in sterile water and sarcosyl (MilliporeSigma, Germany) was added to a final concentration of 1%. The mixture was then incubated for 1 h at room temperature with continuous rotation to remove the inner membrane proteins. The samples were subsequently ultracentrifuged for 1 h at 100,000 × g. Finally, the pellets were collected and washed three times with sterile water. The resulting pellet, rich in OMPs, was resuspended in sterile water. The total protein concentration of each sample was then measured using the Bradford assay (Bio-Rad, Hercules, CA, USA). Equal amounts of the prepared OMPs (30 μg) were separated by SDS-PAGE and gels were stained by coomassie blue to visualize the OMP profiles. We selected an estimated <75 kDa band that appeared to be strongly upregulated upon the growth of *A. baumannii* A118 in LB + 10% serum. Selected protein band was excised from the SDS gel, protein was eluted and identified by LC–MS/MS (Texas Tech University Center for Biotechnology and Genomics, Lubbock, TX). For data processing, Proteome Discoverer (Ver. 2.4) (Thermo Scientific, MA, USA) was used to generate peptide mass spectra, mass list, coverage percentage and Score Sequest HT.

### 2.10 Intracellular metal ions measurement using ICP-MS

The samples were prepared for ICP-MS analysis using a previously established protocol with minor modifications (Wakeman et al., 2014). Bacterial serum-treated cultures were collected, pelleted, and washed in 500 μL of Chelex-treated 1X phosphate-buffered saline (PBS) at two time points (4 and 8 HPI). Three biological replicates were collected for each time point. The pellet was resuspended in 1 mL of Chelex-treated, metal-free water and sonicated at 100% amplitude for 30 s per sample. To prevent cross-contamination, the sonication probe was thoroughly cleaned between samples by wiping and rinsing with Chelex-treated water. To monitor for potential metal contamination during the process, a water control was sonicated after every two samples. Protein concentrations were normalized across samples, and 100 μL of each sample was digested by adding 1 mL of 50% Optima-grade nitric acid (Fisher Scientific). Digestion was performed overnight at 50°C in metal-free 15-mL conical tubes. Following digestion, samples were filtered (0.22 μm, nylon, VWR), and gravimetrically diluted as needed so that the concentrations of target elements fell within the calibration range (0 to 500 ppb) of the inductively coupled plasma mass spectrometer (ICP-MS) (Agilent 7,900). Concentrations of magnesium (Mg), zinc (Zn), copper (Cu) and potassium (K) were measured in helium mode while iron (Fe) was measured in hydrogen mode. Quality analysis and quality control included the use of rhodium (Rh-103) and indium (In-115) as internal standards, as well as blanks and continuing calibration checks which were run with every batch of 10 samples. Calibration standards were prepared in 15 mL polypropylene tubes (VWR) with 1% HNO3. Detection limits for the quantified elements were estimated using the lowest calibration standard with 80 to 120% recovery: Fe = 0.5 ppb, Mg = 1.0 ppb, Zn = 0.1 ppb, Cu = 0.1 ppb, K = 1.0 ppb. Internal standard recoveries were 87 to 92% and continuing calibration check recoveries were in the range 92 to 116%. Each metal ion measurement from the biological replicates was normalized by subtracting the corresponding blank value for that specific ion.

### 2.11 *Galleria Mellonella* infection model

*Galleria mellonella* infection assay was performed as previously described (Gaddy et al., 2012) with some modifications. Larvae weighing 200 to 400 mg that were kept on wood chips in the dark at 4°C, were used for this assay. *A. baumannii* A118 was cultured in either LB or LB + 10% serum for 8 h. Bacterial cultures were diluted in 1X PBS and adjusted to a concentration of 1 × 10^5^ CFU/mL based on the OD600. The bacterial inocula were verified by plating serial dilutions on LB agar and counting CFU after overnight incubation at 37°C. Each experiment was conducted six times with 10 larvae per group, totaling 60 *G. mellonella* larvae for each condition. Each larva was injected with 10 μL of either sterile 1X PBS or a standardized bacterial culture from LB or LB + 10% serum at the last left proleg and incubated at 37°C in sterile Petri dishes, with viability monitored every 24 h for a total of 120 h. Larvae were evaluated based on their color and by gently prodding them with a glass rod; those that showed no response were classified as dead. Survival curves were generated using the Kaplan–Meier method (Kaplan and Meier, 1958) using GraphPad Prism (GraphPad software, San Diego, CA, USA).

### 2.12 Twitching motility

1.5% agar LB plates with and without 10% pooled human serum, were prepared fresh for each experiment. Each twitching motility plate was stab inoculated with a colony of bacteria at the agarose/petri plate interface and placed in a 37°C, humidified incubator for 48 h. To visualize the bacteria at the interface, agarose was removed from each plate and the opaque zone of bacterial movement on the plastic surface was stained by 0.1% crystal violet for 10 min. Then plates were washed several times with DI water until the rinse ran clear.

### 2.13 Statistical analysis

Statistical analyses and graphical plotting of all experiments results except RNA-Seq analysis were conducted using R 4.2.1 and GraphPad Prism 9.5.1 (GraphPad Software, Inc., San Diego, CA). One-way ANOVA followed by Tukey’s multiple-comparison test was used to assess statistical significance of ICP-MS and biofilm formation data.

## 3 Results

### 3.1 WBBP exposure leads to repression of acinetobactin biosynthesis and transport and upregulation of nitrate assimilation pathways in *Acinetobacter baumannii* A118

We analyzed the transcriptomic changes of *A. baumannii* A118 grown in WBBP compared to WBHV, with each condition including samples from three patients or healthy volunteers. In the PCA plot, PC1 accounts for 33.65% of the total variance, representing the largest source of variation in the dataset and indicating a moderate but meaningful degree of separation among the samples (Supplementary Figure 1). RNA-Seq analysis comparing *A. baumannii* A118 grown in WBBP versus WBHV identified 118 DEGs (padj ≤ 0.05, |log2FoldChange| ≥ 1), with 59 genes upregulated and 59 downregulated under the WBBP condition (Supplementary Figure 2; Supplementary Table 1).

**Figure 1:**
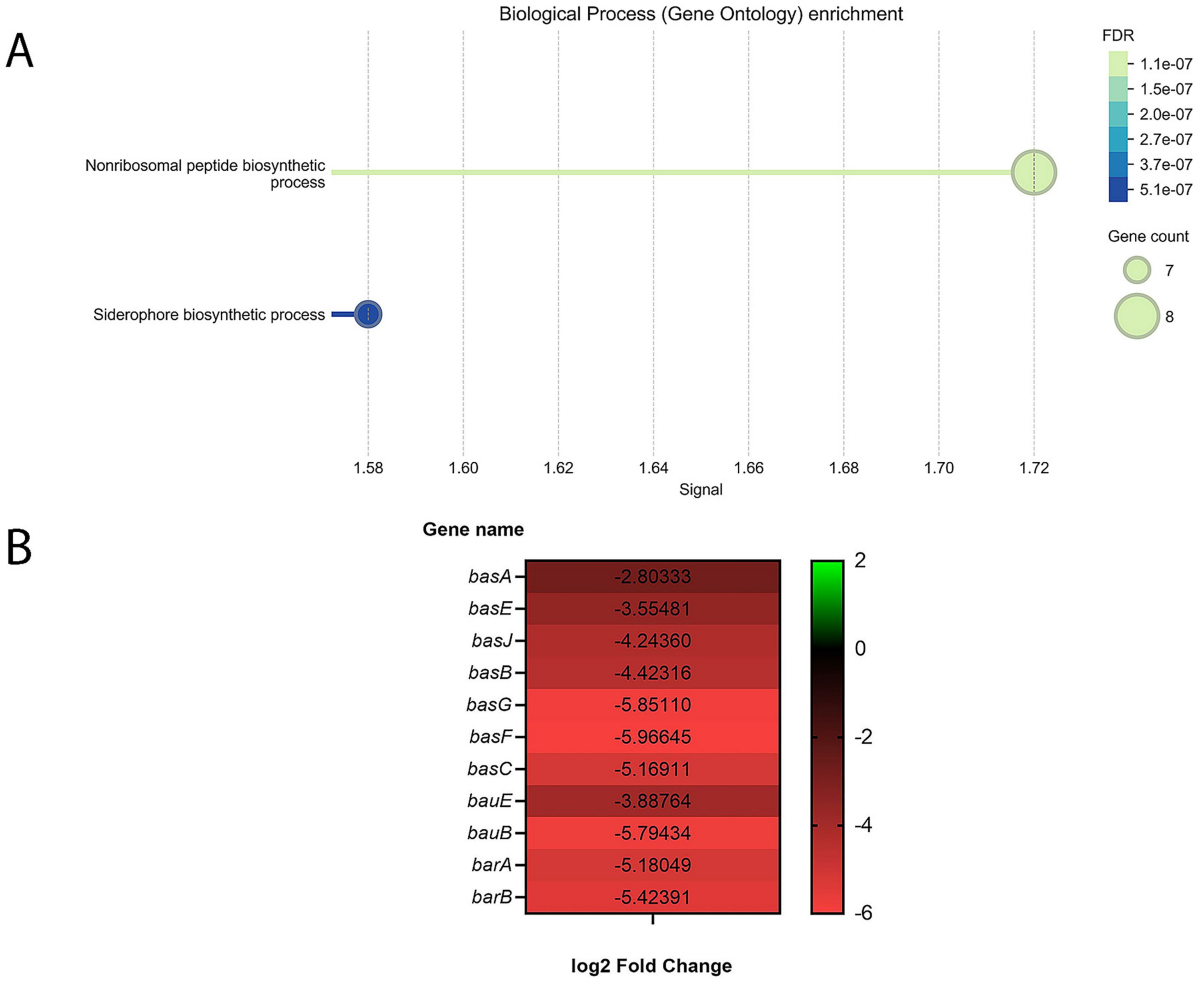
**(A)** GO enrichment analysis of *A. baumannii* A118 genes downregulated in response to WBBP compared to WBHV, within the Biological Process category. The X-axis represents the Signal value, and the Y-axis shows the GO term descriptions. GO terms were filtered using the following criteria: Term similarity ≥0.70, FDR ≤ 0.05, Signal ≥0.50, Strength ≥0.25, and Minimum count in the network was set to 10. **(B)** Heatmap showing 11 out of 18 acinetobactin operon genes downregulated in *A. baumannii* A118 in response to WBBP compared to WBHV (padj ≤ 0.05 and |log2foldchange| ≥ 1).

GO analysis of downregulated genes revealed that, two GO terms within the biological process category were significantly enriched under the WBBP condition, both showing substantial overlap (Figure 1A). According to STRING’s GO analysis, the nonribosomal peptide biosynthetic process term had the higher signal value and STRING assigned a total of 17 genes—within our network and the background—to this term. Eight downregulated genes from our DEGs were associated with this term (Supplementary Figure 3). The second enriched GO term—siderophore biosynthetic process—includes 7 of the same aforementioned 8 genes—excluding only gamma-glutamyltransferase—and 14 genes in total were annotated with this term based on the STRING database.

The presence of multiple acinetobactin biosynthesis and transport genes within both GO terms led us to manually examine the full list of downregulated genes to better understand how WBBP affects the production and transport of this siderophore. Out of the 18 genes in the acinetobactin operon, 11 were found to be downregulated in our dataset (Figure 1B).

Although STRING GO analysis did not reveal a significantly enriched GO term among the upregulated genes, it identified a local network cluster labeled: “Nitrate assimilation and nitronate monooxygenase activity” (FDR = 0.0087, signal = 0.67, count in network = 4 of 6). This network cluster highlights two of the most highly upregulated genes within our DEGs: *nirB* (nitrite reductase large subunit, log2FoldChange = 4.48) and *nirD* (nitrite reductase small subunit, log2FoldChange = 4.04). The other two genes in this network cluster—*nasA* (nitrate reductase, log2FoldChange = 2.42) and a nitrate/nitrite transporter (log2FoldChange = 3.23)—were also among the most highly upregulated DEGs, further supporting the activation of nitrate assimilation pathways in response to WBBP (Supplementary Table 1).

### 3.2 Human serum impacts the growth dynamics of *Acinetobacter baumannii* A118

To better understand the effects of specific components of the human blood that would influence *A. baumannii* A118 gene expression, we also analyzed transcriptomic changes in response to 10% human serum added to LB. While the whole blood experiment provided insight into host-pathogen interactions in physiologically relevant but complex ex vivo conditions, the LB + 10% serum model allowed us to isolate and investigate the effects of serum-derived signals in a more defined environment. By comparing these datasets, we aimed to distinguish between general serum-driven responses and those specific to the altered immune/nutritional status of burn patients.

**Figure 2:**
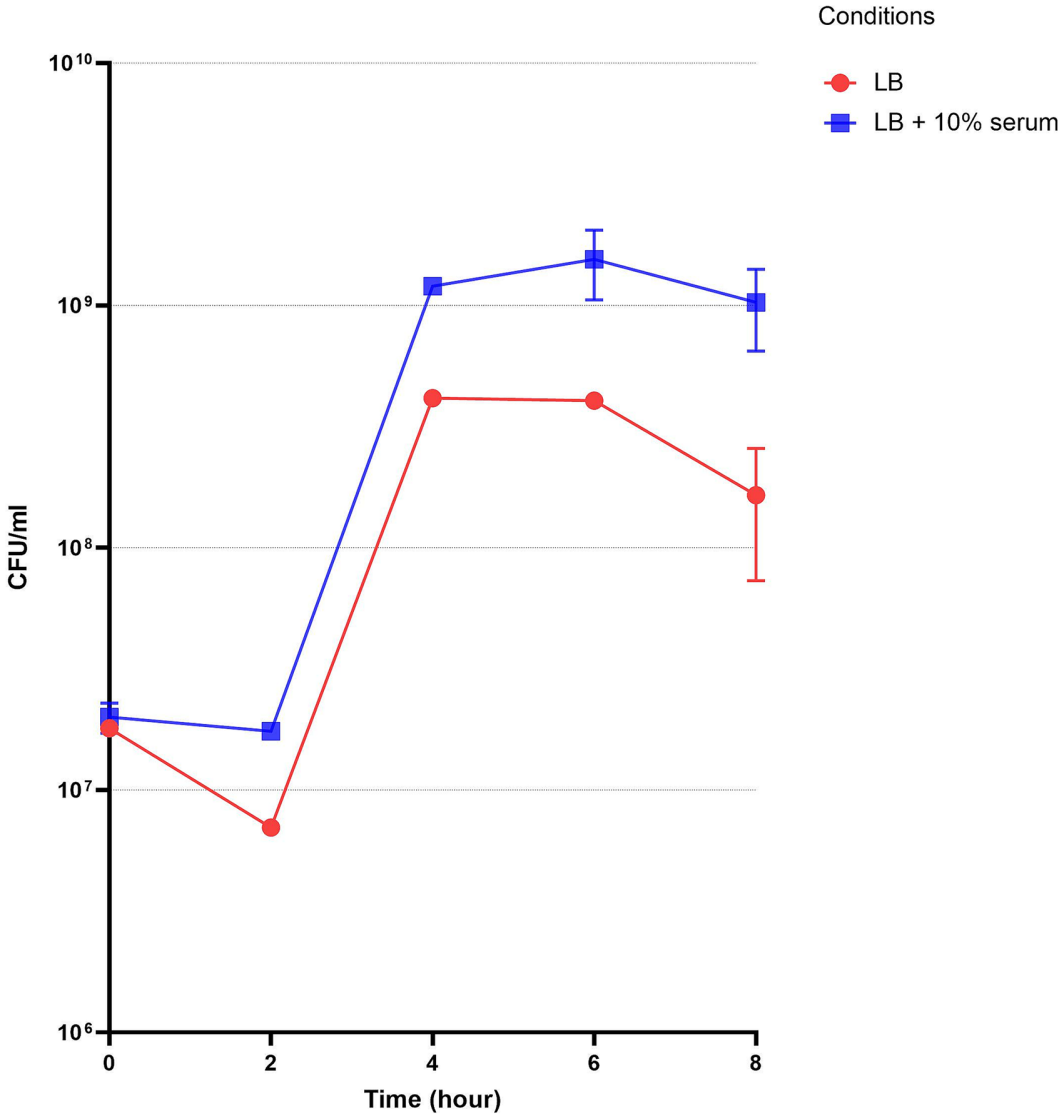
Standard growth curve of *A. baumannii* A118 grown in LB or LB + 10% serum over 8 h. CFU counts were measured every 2 h from three replicates per group.

The initial evaluation of serum’s effect on *A. baumannii* A118 involved assessing its standard growth curve over an 8-h period. The growth curve analysis was conducted in both LB and LB + 10% serum (Figure 2). The initial bacterial inoculum for both media was similar and around 2–1.8 × 10^7^ CFU/mL. As the incubation continued, significant differences in growth between the two conditions emerged. At the 2 HPI, the CFU/ml in LB decreased to 7.0 × 10^6^ CFU/mL, while the decline in LB + 10% serum, was more moderate, reaching 1.75 × 10^7^ CFU/mL. This period might represent an adaptation phase, typically showing a reduction from the starting inoculum. Therefore, the addition of human serum appears to have attenuated this decline compared to pure LB.

By 4 HPI, bacterial growth in LB reached 4.15 × 10^8^ CFU/mL, while that in LB + 10% serum was significantly increased reaching 1.2 × 10^9^ CFU/mL. The period between this point and that at 6 HPI, may represent a short stationary phase. At the 6 HPI, the bacterial concentration in LB broth dropped slightly to 4.05 × 10^8^ CFU/mL, while the presence of serum resulted in a slightly higher concentration—1.55 × 10^9^ CFU/mL—compared to 4 HPI point of this condition.

At the 8 HPI, the bacterial concentration in LB decreased to 1.65 × 10^8^ CFU/mL, indicating a decline in growth, while in LB + 10% serum, the CFU/ml only slightly decreased to 1.03 × 10^9^. While the exact timing of the growth phases may vary between the two conditions, our data demonstrates that compared to growth in LB alone, the addition of human serum significantly enhances the overall growth of *A. baumannii* A118.

### 3.3 Time-dependent effects of human serum on *Acinetobacter baumannii* A118 transcriptome reveal greater impact at later growth stages

To gain a deeper understanding of how human serum influences gene expression in *A. baumannii* A118 and the potential role of the growth phase in this influence, we compared bacterial transcriptomic changes at two distinct time points in response to human serum. We selected 4 HPI, which represents the peak of rapid and logarithmic growth in both conditions, and 8 HPI, at which both cultures are likely in the mid-late stationary phase. For each condition and each time point, we examined three biological replicates. Importantly, we obtained the time-points samples at 4 and 8 HPI from the same flask (Supplementary Figures 4, 5). At 4 HPI, the PCA showed a PC1 value of 71% (Supplementary Figure 4), however by 8 HPI, the separation between the LB and LB + 10% serum conditions became more pronounced, with PC1 increasing to 75% (Supplementary Figure 5). This greater variance suggests stronger transcriptional divergence over time. Typically, a higher PC1 value correlates with a larger number of DEGs, which is consistent with our findings.

At 4 HPI, RNA-Seq analysis revealed 197 DEGs between LB and LB + 10% serum (padj ≤ 0.05, |log2foldchange| ≥ 2), with 63 genes upregulated and 134 downregulated in the presence of serum (Supplementary Table 2). In contrast, at 8 HPI, the number of DEGs nearly doubled to 386, with 144 upregulated and 242 downregulated (Supplementary Table 3). These results indicate that the influence of human serum on gene expression becomes more pronounced as bacterial growth progresses, leading to broader transcriptional reprogramming at later stages.

### 3.4 During logarithmic phase of growth, human serum induces iron-scavenging and downregulates transport systems

**Figure 3:**
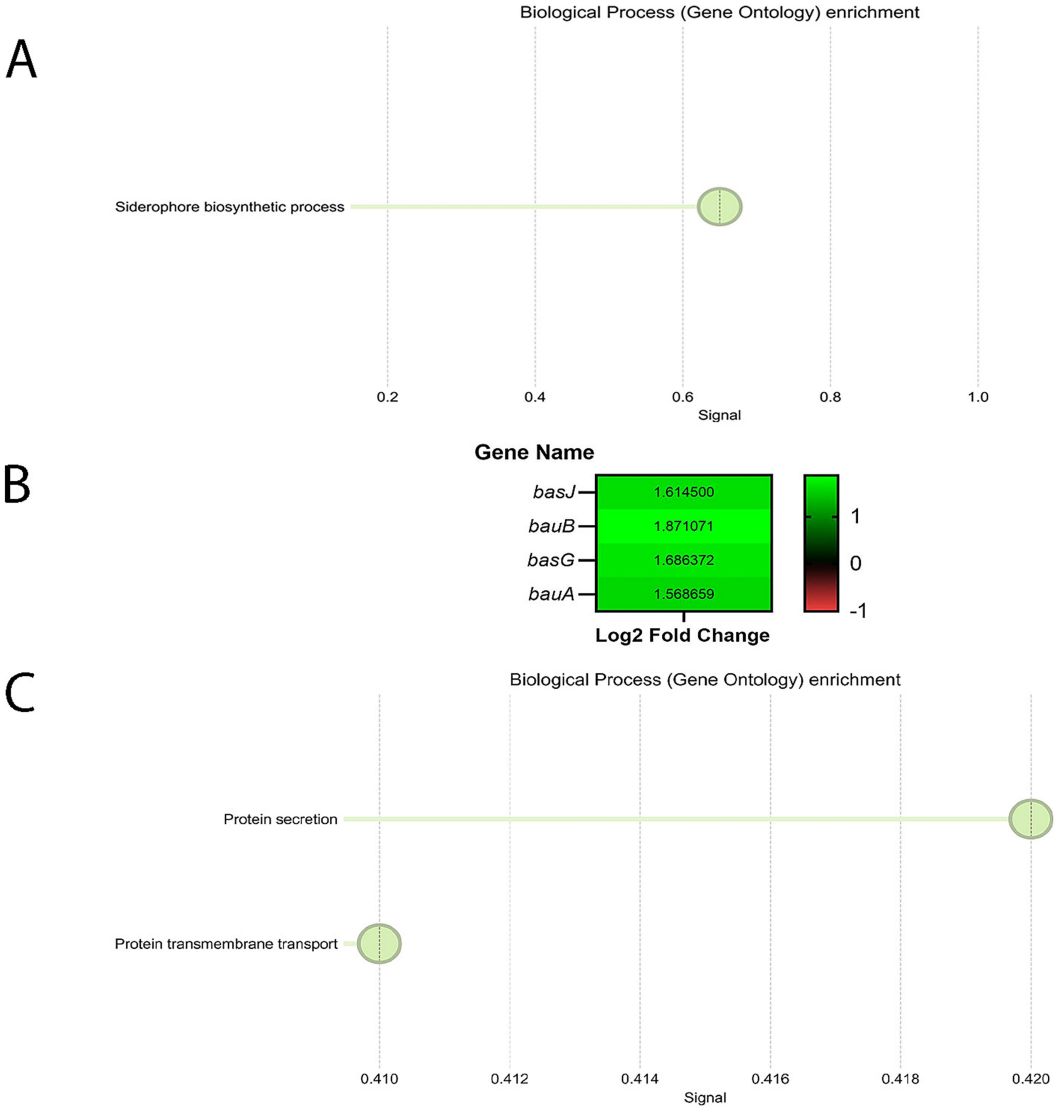
**(A)** GO enrichment analysis of *A. baumannii* A118 genes upregulated in response to serum at 4 HPI, within the Biological Process category. The X-axis represents the Signal value, and the Y-axis shows the GO term descriptions. GO terms were filtered using the following criteria: Term similarity ≥0.70, FDR ≤ 0.05, Signal ≥ 0.50, Strength ≥ 0.25, and Minimum count in the network was set to 10. **(B)** Heatmap showing 4 out of 18 acinetobactin operon genes upregulated in *A. baumannii* A118 in response to serum at 4 HPI (padj ≤ 0.05 and |log2foldchange| ≥ 1). **(C)** GO enrichment analysis of *A. baumannii* A118 genes downregulated in response to serum at 4 HPI, within the Biological Process category. The X-axis represents the Signal value, and the Y-axis shows the GO term descriptions. GO terms were filtered using the following criteria: Term similarity ≥0.70, FDR ≤ 0.05, Signal ≥ 0.50, Strength ≥ 0.25, and Minimum count in the network was set to 10.

To identify pathways affected by serum at both time points, we performed STRING GO analysis on DEGs from our RNA-Seq data. At 4 HPI, comparing LB + 10% serum to LB, we detected significant enrichment in the biological process category for both up- and down-regulated genes (Figures 3A,C). Among the upregulated genes, the only enriched GO term was siderophore biosynthetic process (5 of 14 genes in the network according to STRING; Figure 3A), which was consistent with our manual search of all 18 genes present on acinetobactin operon. We found 4 out of these 5 identified genes among 18 genes present on acinetobactin operon (Figure 3B). The fifth gene from this GO term is annotated as siderophore biosynthesis protein (protein ID: WP_168726283.1). Among the downregulated genes, two GO terms were significantly enriched—protein secretion and protein transmembrane transport— with 9 of 42 and 9 of 48 genes in the network, respectively (Figure 3C). Together, these findings suggest that serum exposure does not cause a broad response at 4 HPI but rather triggers a specific reprogramming of pathways important for host adaptation—especially iron acquisition—while deprioritizing others. It reflects a calculated and efficient early adaptation strategy to host-derived signals.

### 3.5 Exposure to human serum reduces susceptibility of *Acinetobacter baumannii* A118 to several antibiotics

The notable downregulation of GO terms related to protein secretion and transmembrane transport at 4 HPI in response to human serum suggests a potential shift in membrane permeability or transporter activity that might affect antibiotic import/export. Such changes could directly influence the bacterium’s susceptibility to antibiotics. Many antimicrobials, such as Imipenem and Rifampicin, rely on specific outer membrane proteins or active transport mechanisms to enter bacterial cells. Similarly, the activity of efflux systems affects intracellular concentrations of antibiotics like Chloramphenicol, Amikacin, and Tobramycin. Therefore, this transcriptomic change prompted us to examine whether the presence of serum influences antibiotic susceptibility by performing MIC assays in MH agar plates versus MH agar plates supplemented with 10% human serum. Human serum was added directly to MH agar plates to ensure continuous exposure during the 18-h MIC assay. This method better reflects serum’s influence on antibiotic susceptibility compared to pre-growing bacteria in serum, which may lose its effect once transferred to serum-free plates.

We observed increased MIC values for all tested antibiotics when bacteria were grown on MH agar plates supplemented with 10% human serum, compared to MH alone, with varying degrees of change (Table 1; Supplementary Figure 6). The most notable change was seen with amikacin, where the MIC increased from 0.75 mg/L to 3 mg/L, indicating a four-fold decrease in susceptibility. Rifampicin also showed a substantial increase in MIC from 1.5 mg/L to 4 mg/L. Imipenem and tobramycin both demonstrated moderate increases from 0.5 to 0.75 mg/L, while chloramphenicol exhibited a smaller change, rising from 0.24 to 0.32 mg/L. These findings suggest that the presence of human serum reduces bacterial susceptibility to a broad spectrum of antibiotics, potentially due to serum-induced changes in membrane permeability and transporter expression.

**Table 1:** Minimal inhibitory concentration (MIC) (mg/liter) changes of *A. baumannii* A118 for chloramphenicol (C), imipenem (IMI), amikacin (AK), rifampicin (RD) and tobramycin (TOB), in response to serum.

Antibiotic	MIC (mg/liter) in:	
	MH agar plate	MH + 10% serum agar plate
IMI	0.50	0.75
AK	0.75	3
RD	1.5	4
C	0.24	0.32
TOB	0.50	0.75

Equal numbers of bacteria, were inoculated onto two types of plates: one containing Mueller-Hinton agar (MH) and the other Mueller-Hinton agar supplemented with 10% human serum (MH + 10% serum). Each antibiotic teste was conducted in triplicates.

### 3.6 *Acinetobacter baumannii* A118 OMP profiles correlates with observed upregulation of acinetobactin related genes at 4 HPI

**Figure 4:**
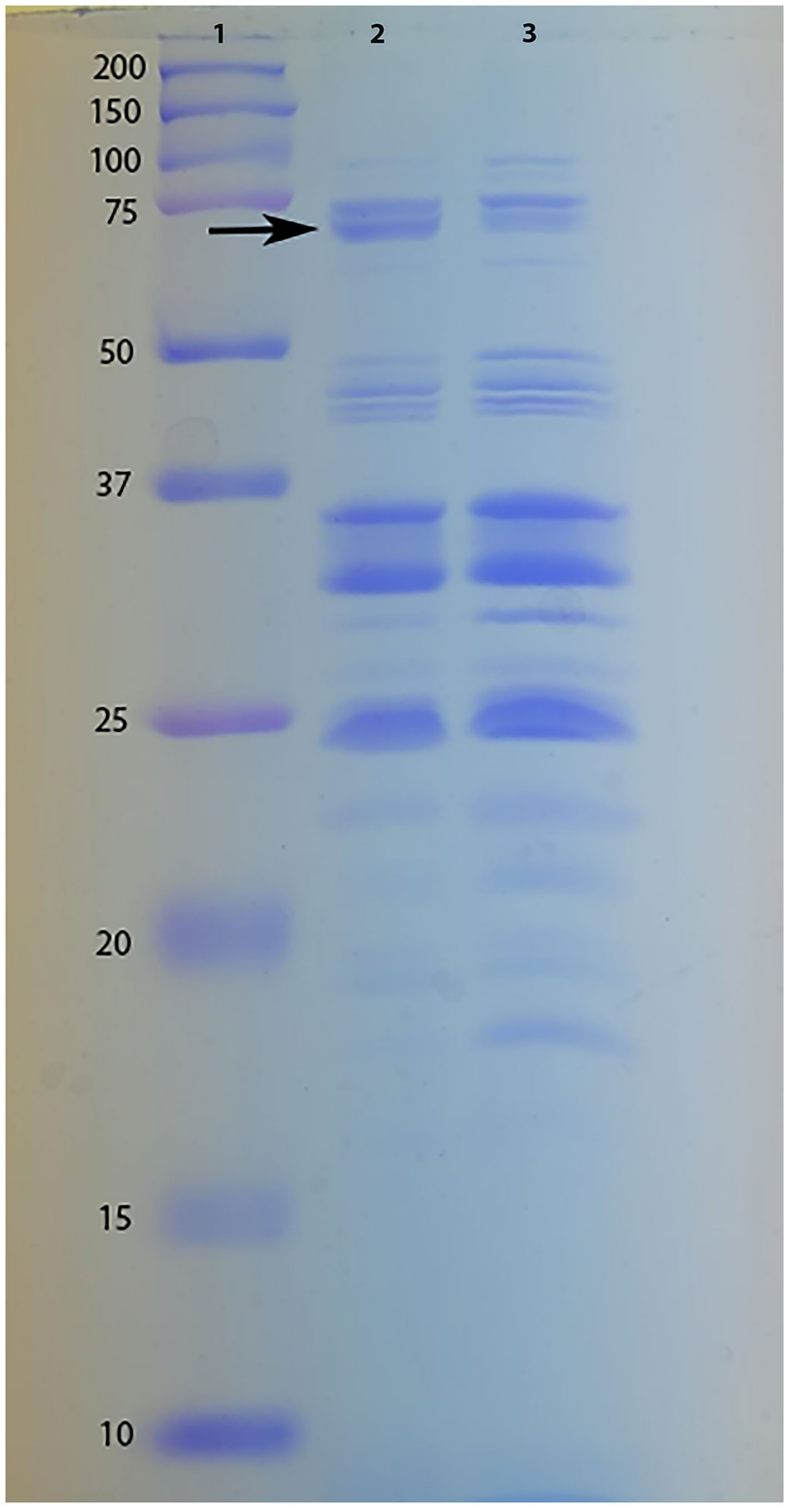
OMP profile of *A. baumannii* A118 grown in LB + 10% serum (lane 2) and LB (lane 3) for 4 h. Lane 1 shows molecular weight standards (kDa). The black arrow indicates the protein band excised from the SDS-PAGE gel and identified by LC–MS/MS.

The downregulation of GO terms related to protein secretion and protein transmembrane transport at 4 HPI and increase MIC values of several antibiotics in response to human serum, led us to hypothesize that the OMP profile of *A. baumannii* A118 grown in LB + 10% serum is potentially different from that in LB alone. To test this, we extracted OMPs from both conditions at 4 HPI and analyzed them via SDS-PAGE, loading equal protein concentrations (Figure 4). Although the overall banding patterns appeared broadly similar between the two conditions, we recognize that SDS-PAGE combined with Coomassie staining lacks the sensitivity and resolution to detect subtle or global shifts in OMP abundance. However, a band just below the 75 kDa marker was visually more prominent in the OMP of bacteria grown in the presence of serum. Therefore, we isolated this band for further analysis and identification. The best match for this band, based on LC–MS/MS analysis, was an outer membrane protein annotated as FepA family TonB-dependent siderophore receptor (Protein ID: WP_228129848.1; molecular weight: 70.6 kDa; number of matched peptides: 15; coverage: 33%; Sequest HT score: 308.77) (Supplementary Table 4). The increase in the production of this protein in the presence of serum correlates with the observed increase in the expression of acinetobactin related genes at 4 HPI.

A manual search of the DEGs at 4 HPI did not identify this gene; however, its presence and relatively high abundance on the SDS-PAGE gel suggests either a post-transcriptional regulation of the acinetobactin operon or a potential increase in the stability of the synthesized protein. Alternatively, this may reflect a temporal disconnect between transcriptomic and proteomic responses, where this gene may be upregulated at earlier time points prior to upregulated at 4 HPI, but the transcriptional level returns to baseline at 4 HPI. However, the protein remains stable and abundant in serum-treated cultures. Additional experiments are required to validate this hypothesis and determine the underlying mechanisms.

### 3.7 Acinetobactin biosynthesis becomes a primary target of downregulation at 8 HPI, marking a shift from earlier induction

**Figure 5:**
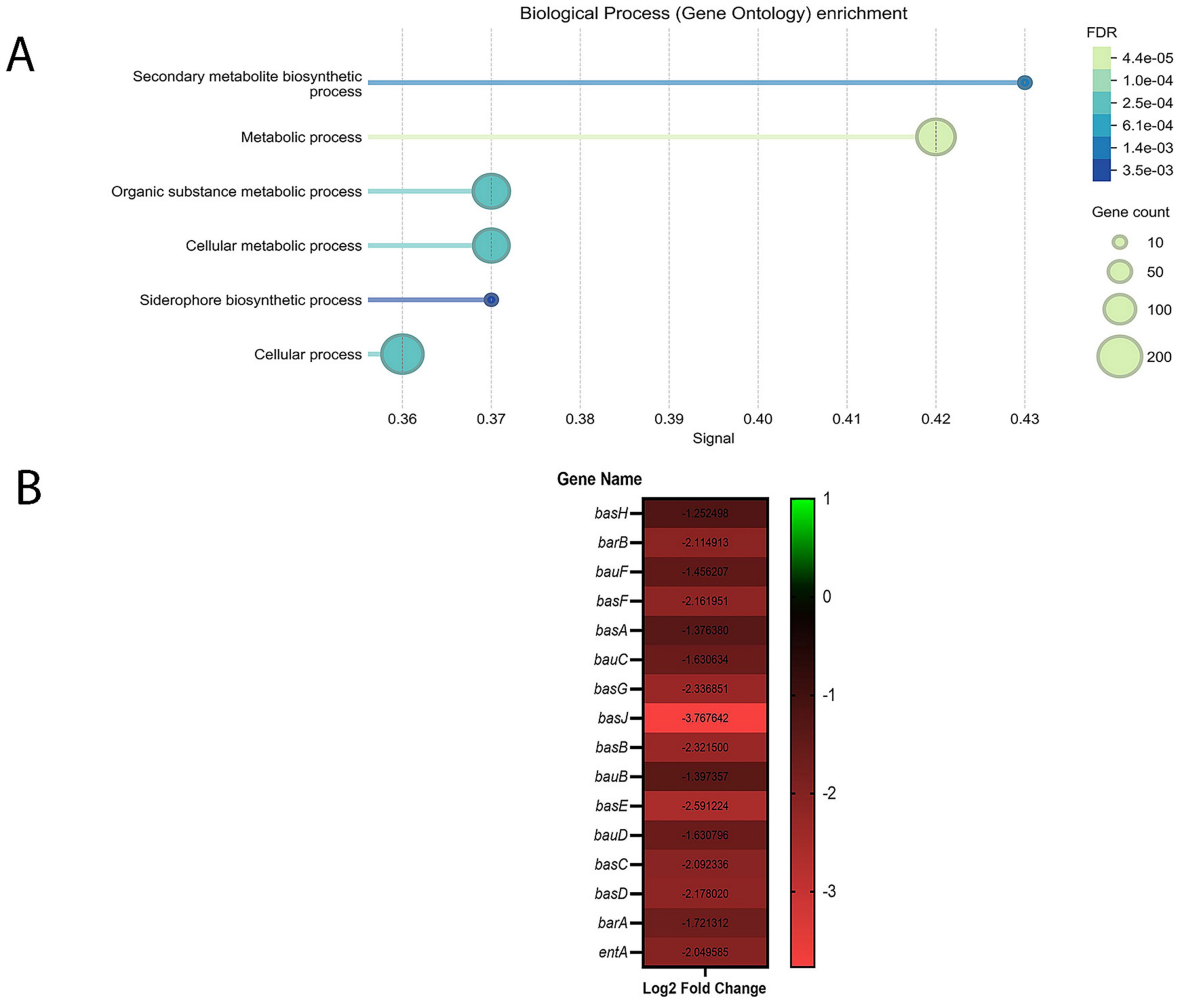
**(A)** GO enrichment analysis of *A. baumannii* A118 genes downregulated in response to serum at 8 HPI, within the Biological Process category. The X-axis represents the Signal value, and the Y-axis shows the GO term descriptions. GO terms were filtered using the following criteria: Term similarity ≥ 0.70, FDR ≤ 0.05, Signal ≥ 0.50, Strength ≥ 0.25, and Minimum count in the network was set to 10. **(B)** Heatmap showing 15 out of 18 acinetobactin operon genes as well as *entA* upregulated in *A. baumannii* A118 in response to serum at 8 HPI (padj ≤ 0.05 and |log2foldchange| ≥ 1).

At 8 HPI, several significantly enriched GO terms were identified among the downregulated genes (Figure 5A), whereas none were found among the upregulated genes at this time point. Interestingly, the GO term siderophore biosynthetic process was significantly downregulated (5 out of 14 genes in the network) in contrast to the 4 HPI time point, where this term was upregulated. Upon manually reviewing the list of DEGs, we found that 15 out of 18 genes in the acinetobactin operon, as well as *entA*, were downregulated at 8 HPI (Figure 5B). *entA* is not located on the acinetobactin operon, however it is essential for the biosynthesis of its precursor. *entA* was not differentially expressed at 4 HPI, highlighting a more pronounced regulatory shift at 8 HPI.

Importantly, the aforementioned GO term significantly overlaps with the top-enriched GO term based on signal value (Figure 5A)—secondary metabolite biosynthetic process—which underscores that the primary target of downregulation at 8 HPI is acinetobactin related.

### 3.8 Serum-induced acinetobactin expression at logarithmic phase, facilitates intracellular accumulation of iron at late stages of growth

Regulation of gene expression related to acinetobactin biosynthesis and transport emerged as a key theme in response to human serum at both time points analyzed. At 4 HPI, we observed upregulation of several acinetobactin related genes in response to serum, with a maximum log2FoldChange of 1.8 (Figure 3B). However, by 8 HPI, not only were more of these genes downregulated, but the extent of downregulation was also more pronounced, reaching log2FoldChange as low as −3.7 (Figure 5B). This pattern led us to hypothesize that during early and logarithmic phases of growth, serum-treated cultures produce high levels of acinetobactin, facilitating iron uptake. As a result, by 8 HPI, bacteria may have accumulated sufficient intracellular iron and, to conserve energy during the transition toward stationary or death phase, began downregulating acinetobactin-related genes. Therefore, we expected higher intracellular iron levels at 8 HPI compared to 4 HPI.

**Figure 6:**
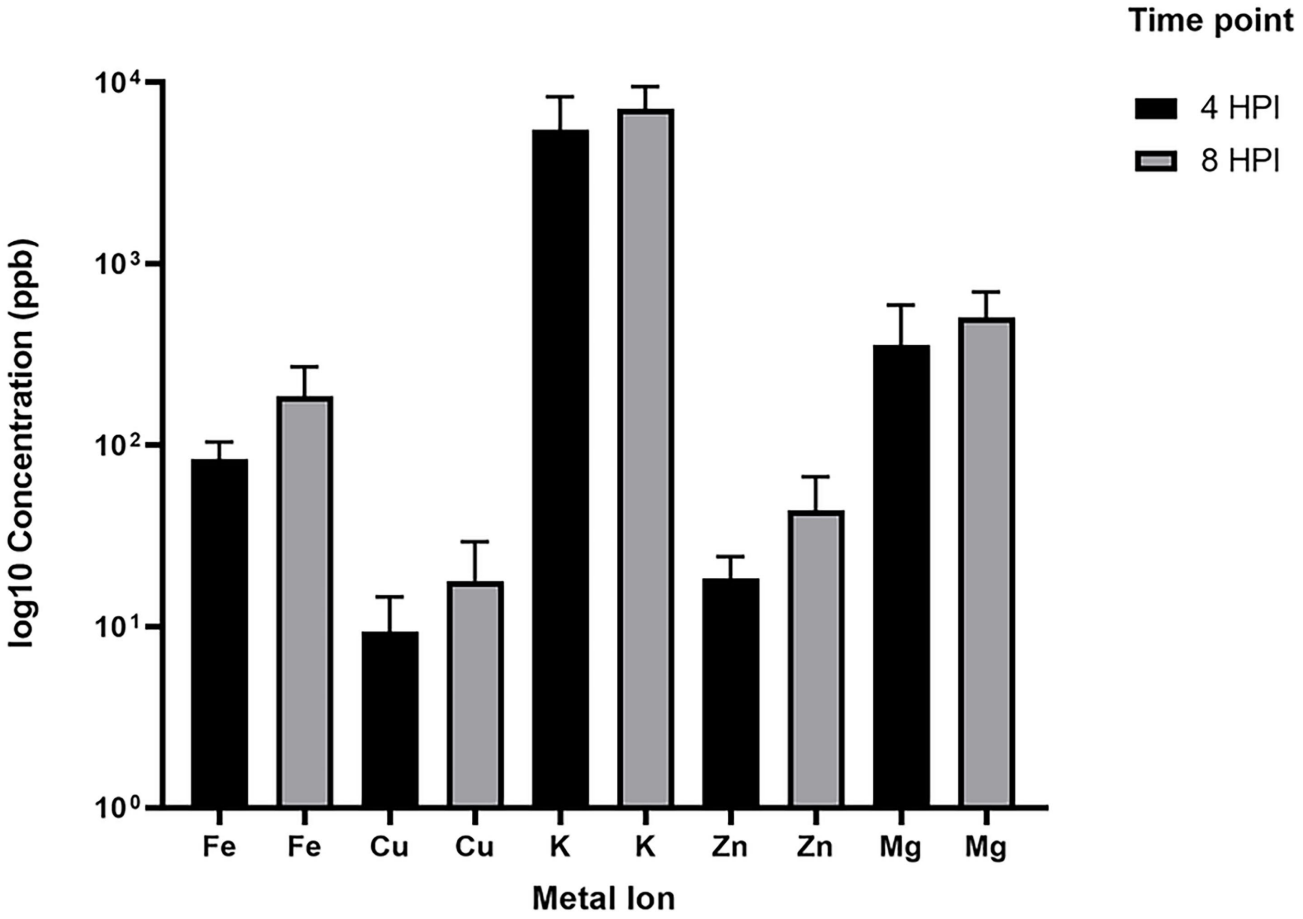
Intracellular levels of all measured metal ions in *A. baumannii* A118 grown in LB + 10% serum were quantified using ICP-MS at 4 and 8 HPI. Error bars represent the standard deviations of triplicate samples.

To test this, we performed ICP-MS analysis on the same serum-treated cultures used for RNA-Seq analysis. Samples were collected at 4 HPI, returned to the incubator, and re-sampled at 8 HPI. ICP-MS results revealed an increase in intracellular levels of several metal ions, including iron, at 8 HPI compared to 4 HPI in serum-treated cultures (Figure 6). While the differences did not reach statistical significance—likely due to limited sensitivity of the instrument causing high variability of the reads between biological replicates— The observed 2.22-fold increase in iron concentration at 8 HPI supports our hypothesis that iron accumulation contributes to the downregulation of acinetobactin genes at this time point. The only other metal ion that showed a fold increase greater than 2 was zinc, with a 2.38-fold increase at 8 HPI.

### 3.9 Human serum enhances twitching motility and promotes biofilm formation by *Acinetobacter baumannii* A118

We observed that several biofilm-associated genes were upregulated at both 4 and 8 HPI in response to human serum (Supplementary Figures 7, 8), indicating a potential role for serum in enhancing the biofilm-forming ability of *A. baumannii* A118. Among the upregulated genes at 4 HPI were those of the *csu* operon, which contribute to early stages of biofilm formation and are also involved in twitching motility. Therefore, we conducted series of experiments to assess the potential effects of serum on both twitching motility and biofilm formation. While our transcriptome analysis was conducted at 4 and 8 HPI, both twitching motility and biofilm formation assays are commonly conducted on cultures grown to 24 or 48 HPI. Thus, it is feasible that a considerable time—24 h or more—is required for proteins encoded by the early regulated genes to produce the observed two phenotypes.

**Figure 7:**
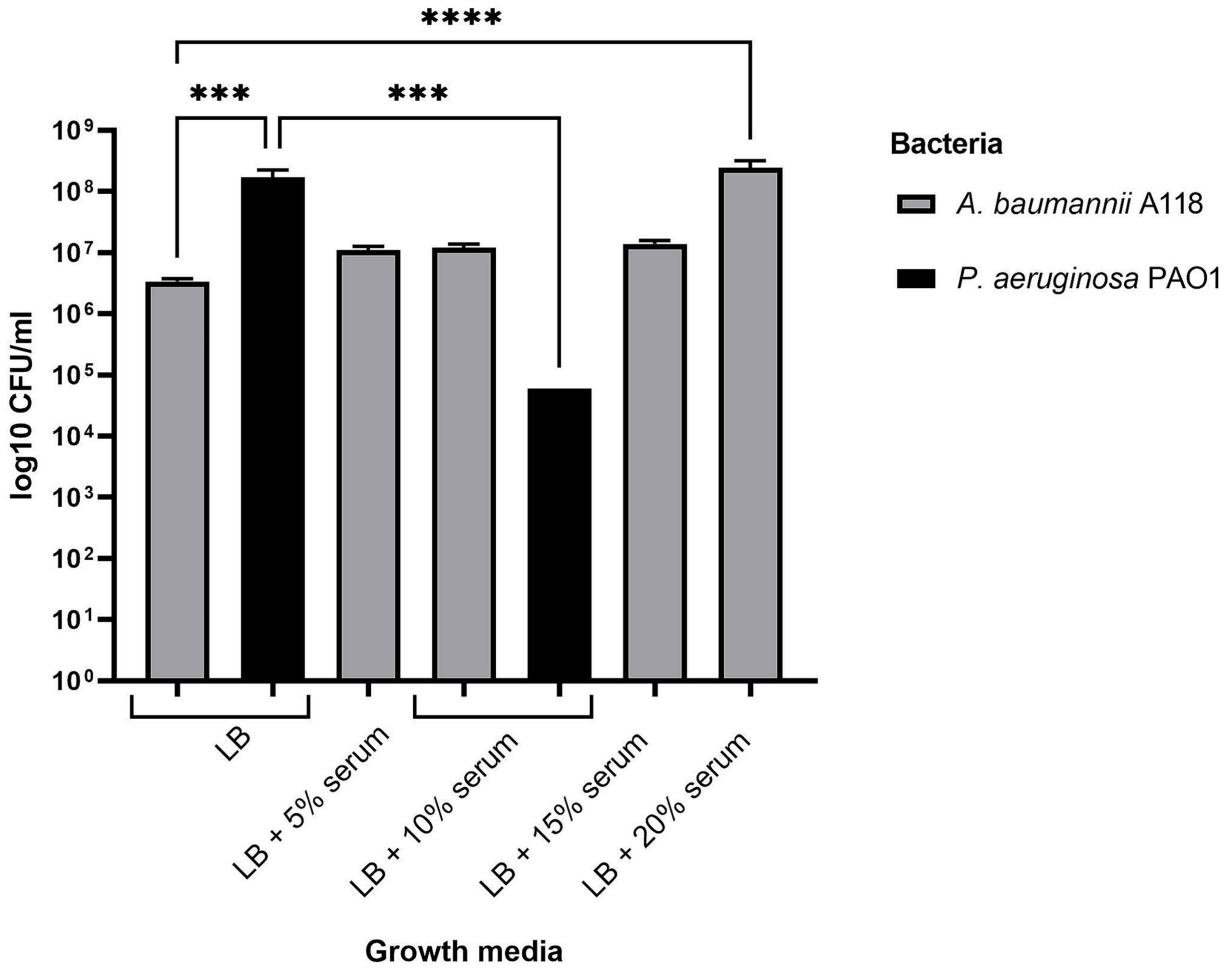
Quantitative analysis of biofilm formation by *A. baumannii* A118 and *P. aeruginosa* PAO1 in LB and LB supplemented with varying percentages of serum. Statistical significance (*p <* 0.05) was determined by one-way ANOVA followed by Tukey’s multiple-comparison test. *p < 0.05; **p < 0.01; ***p < 0.001; ****p < 0.0001.

To test whether serum influences biofilm formation, we first compared biofilm production by *A. baumannii* A118 in LB alone versus LB + 10% serum (Figure 7). While biofilm formation appeared to increase in the presence of 10% serum, the difference was not statistically significant. It possible that at higher concentrations, serum may produce a more prominent effect on biofilm formation. Therefore, we expanded the experiment to test a range of serum concentrations (5–20%). This gradient revealed a consistent, dose-dependent increase in biofilm formation, with statistical significance observed at the 20% serum level. It is possible that this observed effect is not unique to *A. baumannii* A118. Rather, at 20% concentration, serum may enhance biofilm formation by other Gram-negative pathogens. To explore this possibility, we examined the effect of human serum on biofilm formation by *Pseudomonas aeruginosa*— a Gram-negative opportunistic pathogen. We selected PAO1, a well-characterized and virulent *P. aeruginosa* laboratory strain known for its robust and consistent biofilm formation under various laboratory conditions. As expected, PAO1 produced more biofilm than *A. baumannii* A118 in pure LB, and this difference was statistically significant (Figure 7). However, in contrast to its effect *on A. baumannii* A118, the addition of 10% human serum to LB significantly reduced PAO1 biofilm formation by 2.8 × 10^3^-fold. These findings reveal a distinct trait of *A. baumannii* A118 and will be expanded in details in future studies.

These results align with our twitching motility assays, which also showed increased motility in *A. baumannii* A118 when exposed to 10% serum (Supplementary Figure 9), further supporting the hypothesis that serum influences both biofilm formation and twitching motility in this strain.

### 3.10 Serum enhances *Acinetobacter baumannii* A118 *in vivo* virulence, leading to rapid mortality in *Galleria mellonella*

**Figure 8:**
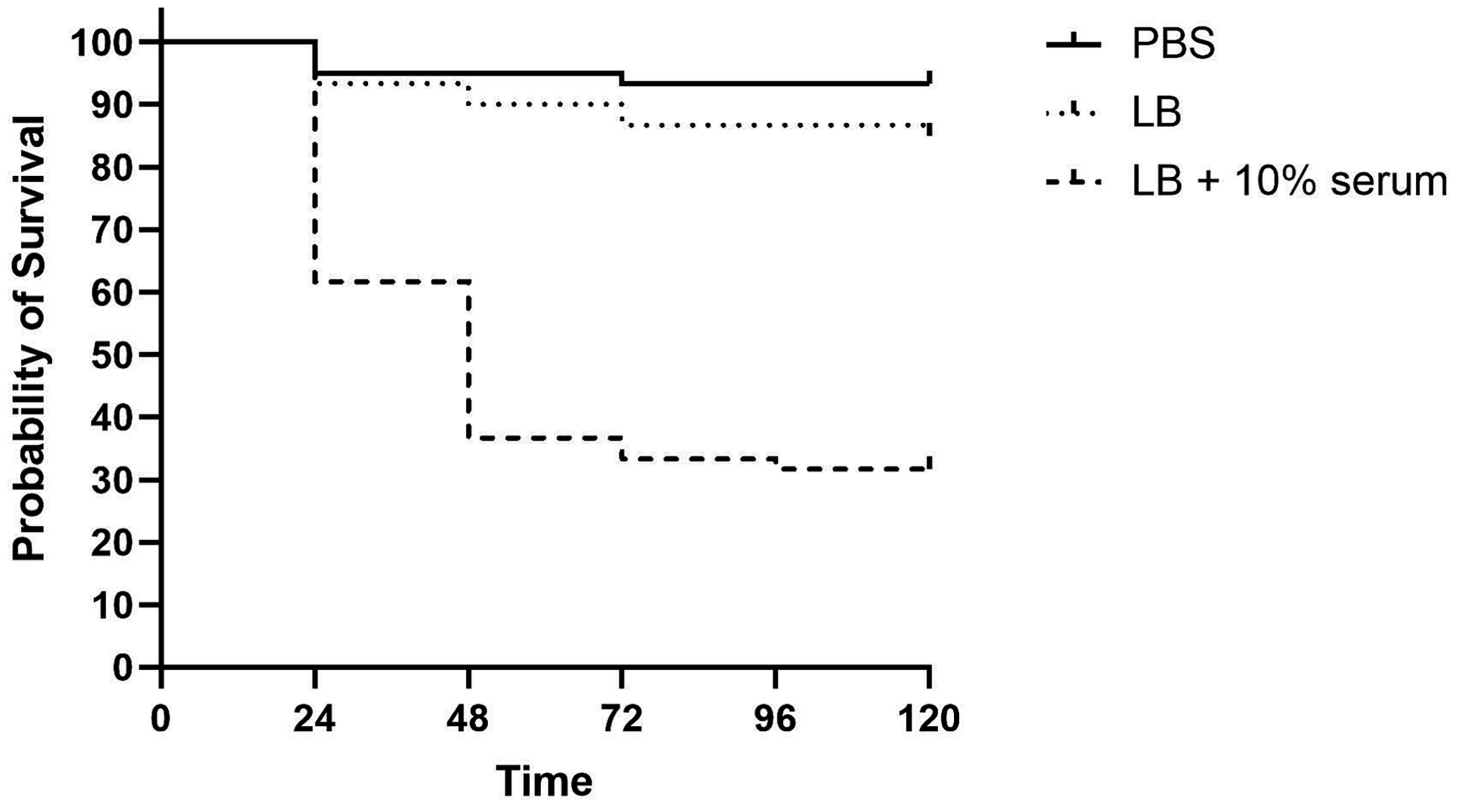
*Galleria mellonella* infection assays showing differences in survival of larvae injected with 10 μL of a standardized suspension of *A. baumannii* A118 at a concentration of 1 × 10^5^ CFU/mL, after growth in either LB or LB + 10% serum, compared to controls injected with sterile 1X PBS.

Our RNA-Seq results and supporting phenotypic assays, all pointing toward serum-induced changes in several bacterial virulence factors. To assess whether these effects translated to a detectable increased in vivo virulence, we used the well-established *G. mellonella* infection model. To capture he full effects of serum, we chose to grow bacteria for 8 h as we observed the biggest DEGs numbers in our 8 HPI RNA-Seq analysis. Larvae were injected with 10 μL of either sterile 1X PBS as the control or standardized bacterial cultures grown in either LB or LB supplemented with 10% human serum. The survival curves over the 5-day period reveal a notable difference (Figure 8). The PBS control group exhibited minimal mortality, maintaining 93.3% survival. Larvae infected with LB-grown bacteria showed a gradual decline to 90% by 48 h and 86.6% by 72 h, remaining stable thereafter. In contrast, larvae infected with serum-exposed bacteria experienced a rapid drop to 61.6% survival within 24 h, decreasing further to 31.6% by 96 h. These results suggest that exposure to serum enhances the virulence potential of *A. baumannii* A118.

## 4 Discussion

Over the past decade, *A. baumannii* has become a significant pathogen in critical care environments, implicated in a wide range of infections including ventilator-associated pneumonia, osteomyelitis, peritonitis, endocarditis, septicemia, meningitis, as well as infections of the skin, wounds, soft tissue and urinary tract (Antunes et al., 2014; Almasaudi, 2018). Given the clinical importance of this opportunistic pathogen and its ability to cause severe infections in immunocompromised individuals—particularly burn patients—there is a noticeable gap in studies examining its gene expression and adaptation in response to the unique environment of whole blood. This gap is especially evident when considering the distinct physiological changes in the blood of burn patients, including during sepsis. Our study aimed to address this underexplored area by investigating the transcriptomic response of a blood-isolated strain of *A. baumannii* grown in WBHV and WBBP, providing new insights into its host-specific adaptation.

We observed upregulation of nitrate assimilation and degradation pathways in *A. baumannii* A118 in WBBP environment, which may be related to the potential increase in plasma nitrate and nitrite in burn patients. Increases in plasma nitrate and nitrite levels have been reported in burn patients, but the criteria and findings vary widely across studies. Some studies found elevated levels in patients with less than 15% TBSA (Gamelli et al., 1995; Harper et al., 1997). However, another study reported decreased levels in that group, with the only observed increase occurring in two patients—one who had 6% TBSA and developed organ failure and sepsis and one who had been treated with compounds containing nitrate (Harper et al., 1997). The wide range of variables across these clinical reports—including patient condition, timing of sample collection, and measurement techniques—suggests that plasma nitrate and nitrite levels may be influenced by multiple factors related to both the clinical course of the burn injury and how samples are processed. While we cannot make any conclusions regarding the plasma nitrate and nitrite levels in our subjects with certainty since we did not measure their levels in our burn subjects, our bacterial response could support previous reports that have observed elevated levels in burn patients.

Among the most downregulated genes in WBBP, those involved in acinetobactin biosynthesis and transport—a key virulence factor in *A. baumannii*—were notably affected. All genes involved in the biosynthesis (*basA*–*J*), efflux (*barAB*), and uptake (*bauA*–*E*) of pre-acinetobactin are encoded from the same locus, except for an *entA*, which is located elsewhere on the chromosome (Sheldon and Skaar, 2020). This system consistently appeared among DEGs and enriched GO terms across all of our transcriptomic analyses, including whole blood and serum experiments. However, the direction of regulation and the number of affected genes within its 18-gene operon varied depending on the specific condition and time of growth. The downregulation of 11 out of these 18 genes in burn patients may be explained by the acute hemolysis of red blood cells (RBCs) commonly observed following thermal injury, particularly within the first week (Loebl et al., 1973). Burns also reduce the lifespan of damaged RBCs, both of which can contribute to elevated levels of free iron in the bloodstream. Further investigation, including biochemical validation and expanded sample collection, will be necessary to confirm this hypothesis.

On the other hand, we observed a mild upregulation trend—specifically, 4 out of 18 genes from acinetobactin operon—in response to 10% serum during the early stages of growth. Interestingly, a much stronger response emerged later, with 15 out of 18 genes, along with *entA*, showing downregulation by 8 HPI. While the significance of the early upregulation remains unclear, we hypothesize that it may support initial iron uptake, leading to intracellular iron accumulation and subsequent downregulation of the operon. This observation prompted us to perform intracellular iron measurements using ICP-MS. While variability among biological replicates limited statistical significance within our ICP-MS data, we observed a consistent trend toward higher intracellular iron levels at 8 HPI compared to 4 HPI in the serum-treated group. This trend might partially support the idea that elevated intracellular iron at later stages may contribute to the observed downregulation of acinetobactin genes. Additional support for our hypothesis came from bacterial OMP extraction at 4 HPI. SDS-PAGE analysis revealed largely similar outer membrane protein profiles between LB and LB + 10% serum conditions. One clearly distinguishable upregulated band in the serum-treated sample was identified as a FepA family TonB-dependent siderophore receptor, supporting the idea of enhanced early iron uptake in response to serum. While minor differences in other bands were noted, a more in-depth analysis would be required to determine their identities and relevance.

The addition of serum to LB led to a gradual increase in biofilm formation by *A. baumannii* A118 from 5 to 20%, with statistical significance observed at 20% (*p* < 0.0001). Pimentel et al. (2021) found that 3.5% human serum albumin as well as 100% human serum inhibited biofilm production in three carbapenem-resistant *A. baumannii* (CRAB) strains. Although our results contradict those of Pimentel et al. (2021) to some extent, it’s important to recognize that our experimental setup, which combines human serum with nutrient-rich LB broth, differs from other studies (Pimentel et al., 2021) that use human serum as the sole medium. Therefore, these studies should be considered separately, taking their specific experimental designs into account. The significance of the growth media can be illustrated by comparing two studies conducted on the same strain, *A. baumannii* AB5075. Martinez et al. (2021) demonstrated a slight increase in biofilm formation when using LB + 4.5% cerebrospinal fluid, which contains human serum albumin as a key component. In contrast, Pimentel et al. (2021) reported a decrease in biofilm formation for the same strain when grown in LB + 3.5% human serum albumin. This comparison highlights how variations in media composition can influence biofilm production in different ways.

Beyond the direct influence of serum on biofilm formation, another perspective that has emerged in the literature links this phenotype to antibiotic sensitivity. is demonstrated by King et al. (2009) reported a negative correlation between biofilm formation and antibiotic resistance, with their two strongest biofilm-producing *A. baumannii* isolates being highly susceptible to most antibiotics. This pattern was also noted by Pimentel et al. (2021) who found that antibiotic-sensitive *A. baumannii* strains tend to be stronger biofilm producers. The strain used in our study, *A. baumannii* A118, is similarly an antibiotic-sensitive strain (Ramirez et al., 2010), which raises the possibility that its susceptibility may contribute to the enhanced biofilm formation observed in response to serum. However, this connection remains speculative and merits further investigation.

*Galleria mellonella* caterpillars are widely used to study host-pathogen interactions with Gram-negative organisms such as *A. baumannii*, offering significant logistical and ethical advantages over mammalian models (Peleg et al., 2009). To the best of our knowledge, the effect of human serum on the virulence of *A. baumannii* A118 has not been previously investigated using this infection model. Our study provides unique and novel insights into how serum influences the overall virulence of *A. baumannii* A118.

## Data Availability

The datasets presented in this study can be found in online repositories. The names of the repository/repositories and accession number(s) can be found in the article/Supplementary material.

## Footnotes

^1^ https://string-db.org/
